# Supplementary material for: Racial and ethnic disparities in benefits eligibility and spending among adults on the autism spectrum: A cohort study using the Medicare Medicaid Linked Enrollees Analytic Data Source
Source: PLoS One. 2021 May 25;16(5):e0251353. doi: 10.1371/journal.pone.0251353 (PMC8148358; doi:10.1371/journal.pone.0251353)
Supplement: S1 Table — (DOCX) [file pone.0251353.s001.docx]

**S1 Table.**

**MMLEADS V2 Source Data Information and Variables**

The MMLEADS V2 2012 data source aggregates Centers for Medicare and Medicaid claims data on three groups of beneficiaries: Medicaid-only with disability, Medicare-only, and beneficiaries who at some point in the year were dual-eligible for both Medicare and Medicaid. The data contain four files that can be merged: Beneficiary file, Condition file, Medicare Services file, and a Medicaid Services file. The Beneficiary file contains information on enrollment and demographics about the beneficiary. The Condition file contains flags for Chronic Condition Warehouse (CCW) conditions that are identified by CMS from a combination of claims data. The Medicare and Medicaid Service files contain monthly and annual utilization and spending metrics aggregated by different utilization types (e.g. “outpatient emergency department visits”). Unlike actual claims data, there are only aggregated counts of utilization and aggregated spending categories that are not tied to specific procedures or diagnosis codes. The utility of this data source is that the aggregated data provide a convenient way to examine broad utilization and cost questions. The data are de-duplicated to remove counts of redundant claims from both Medicare and Medicaid occurring from a single beneficiary claim occurrence. The drawback of this data source is that there is no way to tie utilization to procedure or diagnosis codes related to that utilization or spending, since they are aggregated across multiple claims and multiple source files.

Independent, dependent, and covariate variables included in our analyses included:

| **Original Variable** | **Variable Name** |
| --- | --- |
| e_mme_type | Beneficiary’s annual eligibility status |
| d_medicaid_race | Medicaid Race Code |
| d_medicare_race | Research Triangle Institute (RTI) Race Code |
| d_sex | Sex of the beneficiary |
| 3 separate Chronic Condition Warehouse (CCW) codes per condition listed in the MMLEADS V2 manual. Example: Acute Myocardial Infarction (AMI) variables  *Example:*  ami_medicare  ami_medicaid  ami_combined | CCW Flag for meeting criteria and/or claims for the condition (variable codes are 1 or 3 for any of the three condition flags from Medicare, Medicaid, or both) |
| d_county | County code |
| d_state_cd | Annual state of residence, collapsed into geographic region of residence using Census Bureau geographical regions |
| e_orec | Original reason for entitlement for Medicare |
| e_boe | Basis of eligibility for Medicaid |
| e_waiver1_01 | Medicaid waiver eligibility for January of 2012. There were three waivers documented per beneficiary and this is waiver 1. |
| s_medicare_pmt | Annual Medicare payments for services |
| s_medicaid_pmt | Annual Medicaid payments for services |
| s_medicare_bene_pmt | Annual Medicare beneficiary payments |
| s_medicaid_bene_pmt | Annual Medicaid beneficiary payments |

Summarized from the MMLEADS V2 User Guide (<https://www2.ccwdata.org/documents/10280/19002246/mmleads-user-guide-v2-0.pdf>)
