# Supplementary material for: Racial and ethnic disparities in benefits eligibility and spending among adults on the autism spectrum: A cohort study using the Medicare Medicaid Linked Enrollees Analytic Data Source
Source: PLoS One. 2021 May 25;16(5):e0251353. doi: 10.1371/journal.pone.0251353 (PMC8148358; doi:10.1371/journal.pone.0251353)
Supplement: S2 Table — (DOCX) [file pone.0251353.s002.docx]

**S2 Table.**

|  | **18-24 years** | | **25-34 years** | | **35-44 years** | | **45-54 years** | | **55-64 years** | |
| --- | --- | --- | --- | --- | --- | --- | --- | --- | --- | --- |
|  | **OR** | **SE** | **OR** | **SE** | **OR** | **SE** | **OR** | **SE** | **OR** | **SE** |
| White | Ref |  | Ref |  | Ref |  | Ref |  | Ref |  |
| Black | 0.82* | 0.02 | 0.69* | 0.02 | 0.48* | 0.02 | 0.37* | 0.02 | 0.33* | 0.03 |
| Asian/PI | 0.81* | 0.06 | 0.88 | 0.05 | 0.89 | 0.09 | 0.42* | 0.07 | 0.19* | 0.04 |
| Other/ More than 1 | 1.32* | 0.11 | 1.61* | 0.14 | 1.60* | 0.25 | 0.90 | 0.18 | 1.46 | 0.49 |
| Hispanic | 0.73* | 0.03 | 0.82* | 0.03 | 0.78* | 0.05 | 0.81* | 0.08 | 0.76 | 0.13 |
| Has intellectual disability | 0.83* | 0.02 | 0.76* | 0.02 | 1.22* | 0.05 | 2.91* | 0.15 | 5.57* | 0.38 |
| Has costly chronic condition | 2.34* | 0.06 | 2.03* | 0.04 | 1.76* | 0.06 | 1.51* | 0.07 | 1.52* | 0.10 |
| Female | 0.95 | 0.03 | 0.88* | 0.02 | 0.85* | 0.03 | 0.78* | 0.04 | 0.83* | 0.05 |
| Northeast | Ref |  | Ref |  | Ref |  | Ref |  | Ref |  |
| Midwest | 0.74* | 0.02 | 0.86* | 0.03 | 0.84* | 0.04 | 0.79* | 0.05 | 0.63* | 0.06 |
| South | 0.85* | 0.03 | 0.67* | 0.02 | 0.70* | 0.03 | 0.67* | 0.04 | 0.57* | 0.06 |
| West | 0.63* | 0.02 | 0.65* | 0.02 | 0.68* | 0.03 | 0.66* | 0.05 | 0.62* | 0.06 |
| Rural | 1.06 | 0.03 | 1.06* | 0.03 | 1.16* | 0.05 | 1.07 | 0.07 | 0.96 | 0.08 |
| County Income | 0.999* | 0.00 | 0.999* | 0.00 | 0.999 | 0.00 | 1.00 | 0.00 | 1.00* | 0.00 |
| Constant | 0.40* | 0.03 | 1.13 | 0.11 | 2.79* | 0.28 | 3.47* | 0.65 | 2.17* | 0.43 |
| LR chi^2^ | 1761.60 |  | 2136.16 |  | 861.54 |  | 920.50 |  | 950.75 |  |
| *N* | 56,753 |  | 44,282 |  | 21,534 |  | 19,493 |  | 11,418 |  |
| Log likelihood | -24863.25 |  | -29472. 121 |  | -11926.31 |  | -7042.85 |  | -3510.36 |  |

Abbreviations: OR, Odds Ratio; LR, Likelihood Ratio; SE, Standard Error

**p<.05*

Data Source: MMLEADS V2, 2012. Centers for Medicare and Medicaid. <https://www2.ccwdata.org/documents/10280/19002246/mmleads-user-guide-v2-0.pdf>

Notes: Five separate logistic regressions across five age categories predicting full-dual status (1) compared to Medicaid-only status (0) adjusting for race-ethnicity, gender, intellectual disability status, costly chronic condition, county median income, and Census region, and rurality. Reference group was: White, No ID, Male, No costly chronic condition in past 2 years, Non-rural, and Northeast region.
