# Supplementary material for: Racial and ethnic disparities in benefits eligibility and spending among adults on the autism spectrum: A cohort study using the Medicare Medicaid Linked Enrollees Analytic Data Source
Source: PLoS One. 2021 May 25;16(5):e0251353. doi: 10.1371/journal.pone.0251353 (PMC8148358; doi:10.1371/journal.pone.0251353)
Supplement: S1 Data — (DOCX) [file pone.0251353.s003.docx]

**S1 Data.**

The data used and described in this study are not publicly-available and the authors legally cannot make this data available due to a Data Use Agreement with CMS that prohibits data sharing (DUA#: RSCH-2020-55304). The data used in this study are available for purchase from Centers for Medicare and Medicaid (CMS) following a data use request from the third-party vendor, the Research Data Assistance Center (ResDAC). Researchers seeking to purchase data should visit [www.resdac.org](http://www.resdac.org) for instructions, guidance, and costs of CMS data. The specific CMS data used in this study were extracted by a third-party vendor from the Medicare-Medicaid Linked Enrollees Analytic Data Source, 2012 files. We requested a cohort-specific data purchase of this research-identifiable data using a finder file request that included the following:

All beneficiaries in the 2009, 2010, 2011, or 2012 Condition files that were flagged with the CCW code for "Autism Spectrum Disorders", "Intellectual Disability and Related Conditions" and "Learning Disabilities and Other Developmental Delays":

AUTISM_COMBINED (values=1 or 3)

AUTISM_MEDICAID (values=1 or 3)

AUTISM_MEDICARE (values=1 or 3)

INTDIS_COMBINED (values=1 or 3)

INTDIS_MEDICAID (values=1 or 3)

INTDIS_MEDICARE (values=1 or 3)

LEADIS_COMBINED (values=1 or 3)

LEADIS_MEDICAID (values=1 or 3)

LEADIS_MEDICARE (values=1 or 3)

**Chronic Condition Warehouse Criteria for “Autism Spectrum Disorder” and “Intellectual Disability”**

|  | **Autism Spectrum Disorder** | **Intellectual Disability** |
| --- | --- | --- |
| **ICD-9 codes** | 299.0, 299.00, 299.01, 299.1, 299.11, 299.8, 299.80, 299.81, 299.9, 299.90, 299.91 | 317, 318, 318.0, 318.1, 318.2, 319, 758, 758.0, 758.1, 758.2, 758.3, 758.31, 758.32, 758.33, 758.39, 758.5, 759.7, 759.81, 759.83, 759.89, 760.71 |
| **Claim Identification** | At least one inpatient claim **OR** two other non-drug claims of any service type during the two-year period | |
| **Reference Period Required for Claim Identification** | 2 years | |

Source: <https://www2.ccwdata.org/web/guest/condition-categories>
