# Supplementary material for: Racial and ethnic disparities in benefits eligibility and spending among adults on the autism spectrum: A cohort study using the Medicare Medicaid Linked Enrollees Analytic Data Source
Source: PLoS One. 2021 May 25;16(5):e0251353. doi: 10.1371/journal.pone.0251353 (PMC8148358; doi:10.1371/journal.pone.0251353)
Supplement: Attachment — Submitted filename: Response to Reviewers.docx [file pone.0251353.s004.docx]

In your revised cover letter, please address the following prompts:

a) If there are ethical or legal restrictions on sharing a de-identified data set, please explain them in detail (e.g., data contain potentially identifying or sensitive patient information) and who has imposed them (e.g., an ethics committee). Please also provide contact information for a data access committee, ethics committee, or other institutional body to which data requests may be sent.

b) If there are no restrictions, please upload the minimal anonymized data set necessary to replicate your study findings as either Supporting Information files or to a stable, public repository and provide us with the relevant URLs, DOIs, or accession numbers. Please see <http://www.bmj.com/content/340/bmj.c181.long> for guidelines on how to de-identify and prepare clinical data for publication. For a list of acceptable repositories, please see <http://journals.plos.org/plosone/s/data-availability#loc-recommended-repositories>.

We will update your Data Availability statement on your behalf to reflect the information you provide.

[Note: HTML markup is below. Please do not edit.]

Reviewers' comments:

Reviewer's Responses to Questions

**Comments to the Author**

1. Is the manuscript technically sound, and do the data support the conclusions?

The manuscript must describe a technically sound piece of scientific research with data that supports the conclusions. Experiments must have been conducted rigorously, with appropriate controls, replication, and sample sizes. The conclusions must be drawn appropriately based on the data presented.

Reviewer #1: Yes

2. Has the statistical analysis been performed appropriately and rigorously?

Reviewer #1: Yes

3. Have the authors made all data underlying the findings in their manuscript fully available?

The [PLOS Data policy](https://nam02.safelinks.protection.outlook.com/?url=http%3A%2F%2Fwww.plosone.org%2Fstatic%2Fpolicies.action%23sharing&data=04%7C01%7Ctbenevides%40augusta.edu%7C3e14dcdf11174dc8b45008d8de531100%7C8783ac6bd05b4292b483e65f1fdfee91%7C0%7C1%7C637503795642818429%7CUnknown%7CTWFpbGZsb3d8eyJWIjoiMC4wLjAwMDAiLCJQIjoiV2luMzIiLCJBTiI6Ik1haWwiLCJXVCI6Mn0%3D%7C1000&sdata=HAQPnk1%2FvX32OWMNzPCJFnARAwejdNWPNR%2B9J7qGwm4%3D&reserved=0) requires authors to make all data underlying the findings described in their manuscript fully available without restriction, with rare exception (please refer to the Data Availability Statement in the manuscript PDF file). The data should be provided as part of the manuscript or its supporting information, or deposited to a public repository. For example, in addition to summary statistics, the data points behind means, medians and variance measures should be available. If there are restrictions on publicly sharing data—e.g. participant privacy or use of data from a third party—those must be specified.

Reviewer #1: Yes

4. Is the manuscript presented in an intelligible fashion and written in standard English?

PLOS ONE does not copyedit accepted manuscripts, so the language in submitted articles must be clear, correct, and unambiguous. Any typographical or grammatical errors should be corrected at revision, so please note any specific errors here.

Reviewer #1: Yes

5. Review Comments to the Author

Please use the space provided to explain your answers to the questions above. You may also include additional comments for the author, including concerns about dual publication, research ethics, or publication ethics. (Please upload your review as an attachment if it exceeds 20,000 characters)

1. Reviewer #1: The study described a significant racial and ethnic disparities of Medicare and Medicaid benefits eligibility among adult autism spectrum disorder (ASD). The findings also show that the total medical cost of patients with ASD existed racial and ethnic disparities.

   The study limitations have been well thought of and it will be good to investigate the racial and ethnic disparities further in a population cohort that is covered by other healthcare insurances. This study will provide additional resources for further understanding and evaluation of the influence of the health outcomes from such disparities of patients with ASD for the health care policymakers.

Author response: Thank you for the supportive comments and helpful feedback! We hope that the revisions we made in response to your constructive points, below, enhances the information for interested readers. We do hope that others build on this work.

Questions/concerns:

1. In the Methods, the authors report only 2012 MMLEADS V2 data was used for the study. Why the authors did not use more recently years, such as 2016, 2017? And is there more background information about this data?

Author response: Thank you for this important question. The 2012 MMLEADS V2 was the most recent available data release at the time of purchase (July 2020). Although more recent MAX and Medicare Research Data Files are available for the years noted (2016, 2017), those have not been aggregated into an MMLEADS data release. We have provided readers with this information by adding, “*representing the latest data release for this source*” on p. 5, line 108. We also added to the Limitations section, p. 18, lines 396+ “*Lastly, although this MMLEADS V2 data release (2012) was the most recent available for purchase as of July 2020, the data reflect a period in U.S. during which great shifts in healthcare were occurring. It is important to use this data as a starting place for benchmarking dual-eligible and other adults on the autism spectrum, however, future researchers should consider newer MMLEADS data releases, as they become available.”*

The reviewer requested more information about the MMLEADS data release. Due to space limitations in the text, we have chosen to add this information to the Appendix S1 for interested readers. We hope this provides the reviewer and readers with the important information needed to understand this data source:

*The MMLEADS V2 2012 data source aggregates Centers for Medicare and Medicaid claims data on three groups of beneficiaries: Medicaid-only with disability, Medicare-only, and beneficiaries who at some point in the year were dual-eligible for both Medicare and Medicaid. The data contain four files that can be merged: Beneficiary file, Condition file, Medicare Services file, and a Medicaid Services file. The Beneficiary file contains information on enrollment and demographics about the beneficiary. The Condition file contains flags for Chronic Condition Warehouse (CCW) conditions that are identified by CMS from a combination of claims data. The Medicare and Medicaid Service files contain monthly and annual utilization and spending metrics aggregated by different utilization types (e.g. “outpatient emergency department visits”). Unlike actual claims data, there are only aggregated counts of utilization and aggregated spending categories. The utility of this data source is that the aggregated data provide a convenient way to examine broad utilization and cost questions. The data are de-duplicated to remove counts of redundant claims from both Medicare and Medicaid. The drawback of this datasource is that there is no way to tie utilization to procedure or diagnosis codes related to that utilization or spending, since they are aggregated across multiple claims and multiple source files.*

2. Did all participants were continually enrolled in Medicaid, Medicare, or dually all year?

Author response: Thank you for this question. The variable that we used was an aggregated eligibility variable created by CMS based on monthly enrollment data. We have provided readers with additional information on Page 6, Line 139-148: “*The annual eligibility variable was created by CMS using a published algorithm that identifies any months where the beneficiary had both Medicare and Medicaid coverage; having at least one month with dual coverage resulted in being classified as one of the dual types. …Over 88% of full-dual beneficiaries had 12-months of this coverage type; 93% of Medicaid-only beneficiaries were covered for 12-months; and 92% of Medicare-only beneficiaries were covered for 12-months. We explicitly did not restrict to 12-months of required coverage, since this would exclude people who died during the year, and people who became eligible for dual-status at some point in the year. Since the purpose was to understand factors that contribute to eligibility status (not specific person-month utilization), this intended sample reflects the research purpose.”*

3. Did authors consider those participants with Medicare Part C?

Author response: Thank you for pointing out our confusing language in the description of spending variables. We have revised that section which now clearly states, “*If the beneficiary was a Medicare Managed Care (Part C) beneficiary, only the beneficiary premiums and spending for Part D drugs were available for inclusion in the spending totals* *because encounter claims for Part C were not reported to CMS until 2018.”*

4. In data analysis, the author mentioned that several states (Alaska, Colorado, Hawaii, etc.) were excluded in all models because of large standard errors. I think the potential reason might because of racial and ethnic disparities of ASD diagnosis in different states. (see this article: <https://stacks.cdc.gov/view/cdc/22182>). Therefore, the authors may include all states and categorize them into “Northeast, South, Midwest, and west”.

Author response: Thank you for this feedback. We re-ran the models with the reviewer suggestion using ‘geographic region of residence’, and have updated our data analyses and appendix models, as well as manuscript language. No substantial differences emerged in this new approach, but it did increase the sample size slightly since beneficiaries in those states were not excluded.

5. Living in urban or rural areas also should be considered.

Author response: The reviewer is absolutely correct that rural/urban geographical location could drive eligibility and spending (our two outcome variables). However, when we ran this in our models presented in the first manuscript version, there was significant co-linearity between rural status and specific states in our model (when states were included as a covariate), due to some states being solely or primarily rural. However, since revising the model to include geographic region instead of state, we were able to include rural status without models needing to drop rural status for collinearity. The models presented now include rural status, as suggested by the reviewer.

6. Medical conditions should be considered as an important covariate in logistic regression model.

Author response: Thank you for this helpful comment. We added a chronic condition indicator covariate to the models and the manuscript now states, “*A costly chronic condition indicator was created from variables provided in the original MMLEADS data source. This variable was coded as ‘1, Yes’ if the beneficiary had one of thirteen chronic conditions, according to the Chronic Condition Warehouse algorithm (29). We used the Centers for Disease Control and Prevention to identify the most costly and economically impactful conditions to include in our analyses (31), which included: depression; epilepsy; diabetes; rheumatoid and osteoarthritis; heart disease conditions such as acute myocardial infarction, hypertension, stroke, and congestive heart failure; cancers including breast, colorectal, lung, and prostrate; Alzheimer’s disease; and obesity.”*

7. Was the cost overall cost? I think the authors should add a short paragraph in the method part to carefully describe how to calculate the cost. In addition, if it is possible based on the data available, the authors may specifically analyze for different types of the cost, such as ASD direct or indirect cost, Emergency department cost, hospitalization cost, ect., and see if it has any racial and ethnic disparities.

Author response: Thank you for this important point. We have expanded our Methods to include more detail about how spending variables were calculated for this study, and to provide additional detail to interested readers, we provide the specific variables and definitions in the Appendix to facilitate replication. The variables we present are overall annual spending by Medicare, Medicaid, and the beneficiary. It is important to note that Medicare and Medicare documentation suggests that their available spending variables are just that—spending—and are not costs as most people like to refer to them. The actual costs of the care may not be captured in the claims data, because Medicare and Medicaid only document the amount that was paid for the services, not the amount that was billed. In other words, there are likely costs that are unaccounted for that the hospital or other providers did not recoup, depending on the service. Like any claims data source, the information available is solely based on what the insurer paid, and what they have documented that the beneficiary paid for plans, co-pays, or other co-insurance documented with the claim. The MMLEADS User Guide makes it clear that these are ‘spending’ and not ‘costs’, so we retain this distinction in our paper. Since this point is likely outside the context of a paper, we have only provided this nuanced description for the reviewer.

The reviewer makes an important point about what services were driving overall costs. Unfortunately, the MMLEADS data are aggregated data from Medicaid MAX claims and Medicare claims, but do not provide the granularity about spending for specific types of utilization. This is an important question which we believe future research will need to answer through using claims data, and therefore have added it to the discussion on future research to extend this work.
